# MicroRNA-28-5p Regulates Liver Cancer Stem Cell Expansion via IGF-1 Pathway

**DOI:** 10.1155/2019/8734362

**Published:** 2019-12-01

**Authors:** Qing Xia, Tao Han, Pinghua Yang, Ruoyu Wang, Hengyu Li, Jin Zhang, Xinfeng Zhou

**Affiliations:** ^1^Department of General Surgery, Hua Mei Hospital, University of Chinese Academy of Sciences, Ningbo, China; ^2^Department of Oncology, General Hospital of Northern Theater Command, Shenyang, Liaoning Province, China; ^3^Department of General Surgery, Third Affiliated Hospital of Second Military Medical University, Shanghai, China; ^4^Department of General Surgery, First Affiliated Hospital of Second Military Medical University, Shanghai, China

## Abstract

**Background:**

MicroRNAs (miRNAs) play a critical role in the regulation of cancer stem cells (CSCs). However, the role of miRNAs in liver CSCs has not been fully elucidated.

**Methods:**

Real-time PCR was used to detect the expression of miR-miR-28-5p in liver cancer stem cells (CSCs). The impact of miR-28-5p on liver CSC expansion was investigated both in vivo and in vitro. The correlation between miR-28-5p expression and sorafenib benefits in HCC was further evaluated in patient-derived xenografts (PDXs).

**Results:**

Our data showed that miR-28-5p was downregulated in sorted EpCAM- and CD24-positive liver CSCs. Biofunctional investigations revealed that knockdown miR-28-5p promoted liver CSC self-renewal and tumorigenesis. Consistently, miR-28-5p overexpression inhibited liver CSC's self-renewal and tumorigenesis. Mechanistically, we found that insulin-like growth factor-1 (IGF-1) was a direct target of miR-28-5p in liver CSCs, and the effects of miR-28-5p on liver CSC's self-renewal and tumorigenesis were dependent on IGF-1. The correlation between miR-28-5p and IGF-1 was confirmed in human HCC tissues. Furthermore, the miR-28-5p knockdown HCC cells were more sensitive to sorafenib treatment. Analysis of patient-derived xenografts (PDXs) further demonstrated that the miR-28-5p may predict sorafenib benefits in HCC patients.

**Conclusion:**

Our findings revealed the crucial role of the miR-28-5p in liver CSC expansion and sorafenib response, rendering miR-28-5p an optimal therapeutic target for HCC.

## 1. Introduction

Hepatocellular carcinoma (HCC) is one of the most malignant tumors in the world, especially in Asian countries [1]. Most HCC patients are diagnosed at an advanced stage with lost surgical opportunity [2]. Liver tumor resection, ablation, and liver transplantation are just suitable for patients diagnosed at an early stage [3]. For these patients with advanced liver cancer, there is no good treatment strategy. Sorafenib is the most used first-line targeted drug for advanced HCC patients, while its therapeutic effect is not satisfactory [4, 5]. Multiple studies have explored the intrinsic mechanisms of cancer cells and the extrinsic microenvironmental factors that influence HCC initiation and progression; however, our understanding of these mechanisms remains incomplete.

Increasing evidence shows that liver cancer stem cells (CSCs) participate in the regulation of tumor initiation, progression, recurrence, and drug resistance [6, 7]. Liver CSCs are a small population of liver cancer cells and can be identified by series liver CSC markers, including epithelial cell adhesion molecule (EpCAM), CD24, CD90, CD133, and OV6 [8–12]. It was reported that CD24-positive liver tumor-initiating cells drive self-renewal and tumor initiation through STAT3-mediated NANOG regulation [9]. Numerous studies also show that recurrence and chemoresistance of HCC are due to the existence of liver CSCs [13]. So, it is urgent to explore the underlying mechanism of liver CSCs' propagation.

MicroRNAs (miRNAs) comprise a class of small, noncoding RNAs that regulate RNA silencing and posttranscriptional of gene expression in general by binding to the 3′-UTR of target mRNAs [14]. Deregulation of miRNAs has been involved in a number of human disease, especially human cancers [15]. miRNAs were also reported to be implicated in the regulation of hematopoietic stem cells as well as hematopoietic malignancies [16]. For instance, miR-181b/Notch2 overcomes chemoresistance by regulating cancer stem cell-like properties in NSCLC [17]. Therefore, liver CSC-specific miRNAs might be potential targets for cancer therapy. Previous studies found that miR-28-5p was downregulated in HCC tissues and suppressed tumor proliferation and migration of HCC cells. However, the biological function of miR-28-5p in liver CSCs is unknown.

In this study, we demonstrate that miR-28-5p expression is downregulated in liver CSCs. Functional tests indicate that miR-28-5p deficiency leads to upregulation of liver CSC self-renewal and tumorigenesis. Further mechanism study reveals that IGF-1 is a direct target of miR-28-5p in liver CSCs. More importantly, we find that miR-28-5p plays an important role in the sensitivity of HCC cells to sorafenib. Taken together, our findings demonstrate the critical role of the miR-28-5p in liver CSC expansion and sorafenib response.

## 2. Materials and Methods

### 2.1. HCC Patients' Tissues

Fifty HCC samples were collected from patients who underwent the resection of their primary HCC in the Eastern Hepatobiliary Surgery Hospital (EHBH); detailed clinicopathological features of the patients is described in the online supplementary [Supplementary-material supplementary-material-1]. Patient informed consent was also obtained, and the procedure of human sample collection was approved by the Ethics Committee of EHBH. Four HCC patients' tissues were used for isolated primary HCC cells. Forty HCC patients' tissues were used for analysis the relationship between miR-28-5p and EpCAM, CD24, or IGF-1. Six HCC patients' tissues were used for PDX analysis.

### 2.2. Cell Culture

The patient-derived primary HCC cultures of tumor cells were obtained from fresh tumor specimens of HCC patients described previously [18]. The human primary hepatoma cells were isolated by collagenase perfusion and centrifugation. Briefly, the liver cancer tissues were washed several times in precooled sterile PBS buffer containing double antibodies to remove blood and connective tissue, GBSS-mixed enzyme solution was used for digestion and centrifugation, and the supernatant was discarded; cell activity was detected by trypanosoma blue staining with cell filtrate, with complete medium heavy suspension inoculation after cell count after the package is cultivated in the bottle, at 37°C and 5% CO_2_ environment culture, and then cell morphology was identified.

HCC cell lines Huh7 and HepG2 were cultured in Dulbecco's modified Eagle's medium (DMEM) supplemented with 10% fetal bovine serum (FBS) and 2 mM L-glutamine and 25 *μ*g/ml gentamicin and maintained at 37°C in a 5% CO_2_ incubator. Huh7 and HepG2 were infected with a miR-28-5p sponge or miR-28-5p mimic lentivirus, and their control lentivirus (Ribobio, Shanghai, China) and the stable infectants were screened by puromycin.

### 2.3. RNA Interference

Small interference RNAs (siRNAs) against IGF-1 and NC (negative control) siRNA were synthetized by Ribobio (Shanghai, China). siRNA target sequences are listed in Supplementary [Supplementary-material supplementary-material-1]. The siRNAs were transfected into the hepatoma cells at a final concentration of 200 nM using siRNA transfection reagent according to the manufacturer's instructions (Polyplus, Illkirch, France). The cells were harvested or subjected to further downstream experiments 24-72 hours after transfection. Gene knockdown was validated by western blotting.

### 2.4. Animal Models

All mouse experiments were performed according to the guidelines of the animal care and use committees at Hua Mei Hospital (University of Chinese Academy of Sciences, Ningbo, China). Four- to six-week-old male athymic NOD-SCID or nude mice (SIPPR-BK Experimental Animal Co., China) were housed and fed in standard pathogen-free conditions.

For *in vivo* limiting dilution assay, hepatoma cells were diluted serially to the indicated doses (1 × 10^3^, 5 × 10^3^, 1 × 10^4^, and 5 × 10^4^) and were mixed with matrigel (1 : 1). Then, the mixed cells were injected subcutaneously into NOD-SCID mice (*n* = 6). After two months, the mice were sacrificed and the number of tumors was counted.

For the patient-derived xenograft (PDX) model, primary HCC tumor samples were obtained for xenograft establishment as described previously [19]. Six surgical specimens were collected from HCC patients. They were placed in a clean phosphate buffer saline (PBS) centrifuge tube and transported to the animal center in an ice box (with tissue preservation time of 30 min-60 min). The tumor was immediately divided into tissue pieces of about 0.3 cm × 0.3 cm × 0.3 cm. 70% alcohol was used to disinfect the skin on the right back of the mice, and 0.5% lidocaine was used for local infiltration anesthesia at the transplantation site of the mice. A small incision about 0.3 cm long was cut off with scissors on the back of the right lower limb of the mice, and 1-2 pieces of the divided tumor tissue were sent to the subcutaneous and pressed for about 2 minutes. This is the establishment of the original PDX animal model, called P0 generation. The subcutaneous tumor of P0 mice grew to about 1,000 mm^3^. The tumor was dissected and placed into an aseptic dish. Part of the tissue was placed in 4% neutral formaldehyde solution and fixed. The rest of the tissue was used to segment the tumor to a size of about 0.3 cm × 0.3 cm × 0.3 cm. Five to ten 5-week-old nude mice were transplanted in accordance with the above method, and the PDX animal model of the 1st generation was called P1 generation. The body mass and tumor volume of mice were measured regularly every week, and the tumor growth curve was plotted. When the subcutaneous tumor of P1 generation nude mice grew to a size of about 1,000 mm^3^, the PDX animal model of generations 2, 3, and 4 was established according to this method, which was called generations P2, P3, and P4, respectively. The mice with xenografts were given sorafenib (60 mg/kg) or vehicle daily orally for 30 days (*n* = 5 for each group). Tumor volumes were measured at the indicated time points. All procedures and protocols were approved by the Ethical Committee of Hua Mei Hospital. Tumor volume = (length × width^2^)/2.

### 2.5. Spheroid Assay

The HCC cells were seeded in 96-well ultralow attachment culture plates (Corning Incorporated Life Sciences) (300 cells per well) and cultured in DMEM/F12 (Gibco) supplemented with 1% FBS, 20 ng/mL bFGF, and 20 ng/mL EGF for 7 days. The number of spheroids was counted, and representative views were shown. The results were repeated three times.

### 2.6. In Vitro Limiting Dilution Assay

The HCC cells were seeded in 96-well ultralow attachment culture plates (Corning Incorporated Life Sciences) (2, 4, 8, 16, 32, and 64 cells per well (*n* = 8)) and cultured in DMEM/F12 (Gibco) supplemented with 1% FBS, 20 ng/mL bFGF, and 20 ng/mL EGF for 7 days. The proportion of CSCs was assessed using ELDA software (http://bioinf.wehi.edu.au/software/elda/index.html) [20]. The results were repeated three times.

## 3. Flow-Cytometric Analysis

For CD24- and EpCAM-positive cell sorting, primary HCC patients' cells and HCC cells were incubated with the primary anti-CD24 (Cat. no. ab202073; Abcam) or anti-EpCAM (BioLegend, Inc., San Diego, CA) for 30 minutes at room temperature. The cells were then subjected to flow cytometry using a MoFlo XDP cell sorter from Beckman Coulter (Indianapolis, IN, USA) according to the manufacturer's instructions. The sorted cells from three independent experiments were subjected to real-time PCR assay.

Hepatoma cells were incubated with the primary anti-EpCAM for 30 minutes at room temperature. Flow-cytometric analysis was performed using a MoFlo XDP from Beckman Coulter according to the manufacturer's instructions. The results were repeated three times.

### 3.1. Apoptosis Assay

HCC cells were treated with sorafenib (10 *μ*M) for 48 h, followed by staining with Annexin V and 7-AAD for 15 min at room temperature in the dark. Apoptotic cells were determined by an Annexin VFITC Apoptosis Detection Kit I (BD Pharmingen, San Diego, CA) and flow cytometer according to the manufacturer's instructions. The results were repeated three times.

### 3.2. Luciferase Reporter Assay

The 3′-UTR of IGF1 plasmid and mutation plasmid were described previously [21]. For the luciferase reporter assay, the HCC cells were seeded on 24-well plates and cotransfected using Lipofectamine 2000 (Invitrogen) with 100 ng per well of the resulting luciferase UTR-report vector, 2 ng per well of pRLCMV vector (internal control, Promega), and 20 ng per well of miR-28-5p precursor molecules or control precursor (Applied Biosystems) following the manufacturer's instructions. After 24 h, the cells were lysed, and the relative luciferase activity was assessed with the Dual-Luciferase Assay Reporter System (Promega). The results were repeated three times.

### 3.3. Real-Time PCR

Total RNA was isolated from cells or tissues using TRIzol (Invitrogen) according to the manufacturer's instructions. The purity of RNA was measured with a UV spectrophotometer (NanoDrop ND-1000), and RNA integrity was validated with agarose gel electrophoresis. The extracted RNA was then reverse-transcribed to cDNA with the M-MLV RTase cDNA Synthesis Kit (Promega). Real-time PCR analysis was performed using a SYBR Green PCR Kit (Roche) and LightCycler 480 System (Roche). PCR conditions included 1 cycle at 95°C for 5 minutes, followed by up to 40 cycles of 95°C for 15 seconds (denaturation), 60°C for 30 seconds (annealing), and 72°C for 30 seconds (extension). The specificity of primers was confirmed by melting curves following the reaction. Each sample was measured in triplicate biological replicates. Hsa-RNU6B and *β*-actin were used as endogenous controls for miRNA and mRNA expression, respectively. The primer sequences are shown in supplementary [Supplementary-material supplementary-material-1]. The results were repeated three times.

### 3.4. Western Blotting Assay

Samples were obtained with cell lysis buffer and disposed as we described before [22]. After quantification with bicinchoninic acid (BCA) assay (Weiao, Shanghai, China), we separated each protein through 10% SDS-PAGE and then moved them onto PVDF membranes (Millipore, USA). Then, samples were blocked with 5% nonfat milk. After incubation with primary antibodies and secondary antibodies, protein levels were detected with ImageQuant LAS 4000 (GE Healthcare Life Sciences). The antibodies are shown in supplementary [Supplementary-material supplementary-material-1].

### 3.5. Statistical Analysis

All experiments were performed at least three times. Data were presented as the mean ± SEM. GraphPad Prism (GraphPad Software, Inc., La Jolla, USA) was used for all statistical analyses. Statistical analysis was carried out using a *t*-test or Bonferroni Multiple Comparison Test: ^∗^*P* < 0.05. A *P* value of less than 0.05 was considered statistically significant.

## 4. Results

### 4.1. miR-28-5p Expression Is Reduced in Liver CSCs

**Figure 1:**
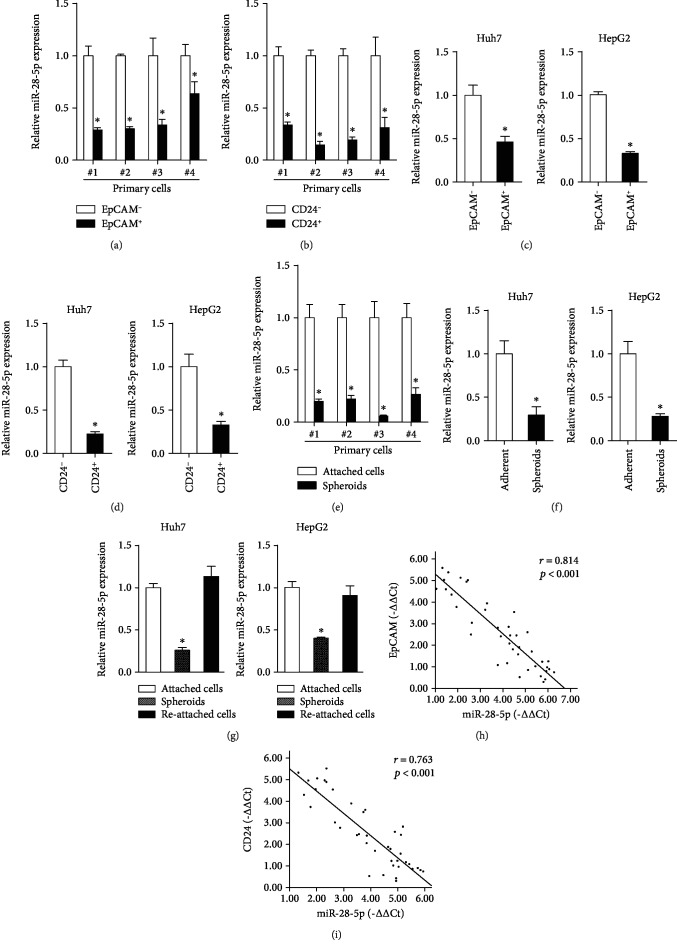
miR-28-5p is downregulated in liver CSCs. (a) EpCAM-positive primary HCC cells and their negative control HCC cells were sorted by flow cytometry and then subjected to RT-PCR assay (*n* = 3). (b) CD24-positive primary HCC cells and their negative control HCC cells were sorted by flow cytometry and then subjected to RT-PCR assay (*n* = 3). (c) Real-time PCR analysis of miR-28-5p in EpCAM-positive HCC cells and EpCAM-negative HCC cells (*n* = 3). (d) Real-time PCR analysis of miR-28-5p in CD24-positive HCC cells and CD24-negative HCC cells (*n* = 3). (e) The expression of miR-28-5p in primary HCC spheroid cells and primary HCC adherent cells was determined by RT-PCR assay (*n* = 3). (f) The expression of miR-28-5p in in HCC spheroid cells and HCC adherent cells was determined by RT-PCR assay (*n* = 3). (g) The expression of miR-28-5p in attached cells, spheroids, and reattached hepatoma cells was determined by RT-PCR assay (*n* = 3). (h) The correlation between the transcription level of miR-28-5p and EpCAM in forty HCC tissues was determined by RT-PCR analysis. Data were normalized to U6 or *β*-actin as *Δ*Ct and analyzed by Spearman's correlation analysis. (i) The correlation between the transcription level of miR-28-5p and CD24 in forty HCC tissues was determined by RT-PCR analysis. Data were normalized to U6 or *β*-actin as *Δ*Ct and analyzed by Spearman's correlation analysis. Data are represented as mean ± s.d.; ^∗^*P* < 0.05; two-tailed Student's *t*-test.

To check the expression of miR-28-5p in liver CSCs, the EpCAM^+^ and CD24^+^ cells were isolated from patient-derived primary HCC cells and HCC cell lines by flow cytometry sorting. As shown in Figures 1(a) and 1(b), miR-28-5p expression was dramatically downregulated in sorted EpCAM^+^ or CD24^+^ primary HCC cells. Consistently, we also found that miR-28-5p expression was decreased in sorted EpCAM^+^ or CD24^+^ HCC cell lines (Figures 1(c) and 1(d)). Moreover, miR-28-5p expression was reduced in HCC spheres derived from human primary HCC cells and HCC cell lines (Figures 1(e) and 1(f)). Furthermore, the miR-28-5p level could be partially restored during reattachment compared with the spheres (Figure 1(g)). More importantly, in HCC tissues, Pearson correlation analysis revealed that miR-28-5p levels were negatively correlated with the expression of CD24 and EpCAM (Figures 1(h) and 1(i)). Taken together, our results showed that miR-28-5p expression was downregulated in liver CSCs.

### 4.2. miR-28-5p Is Responsible for the Maintenance of Liver CSCs

**Figure 2:**
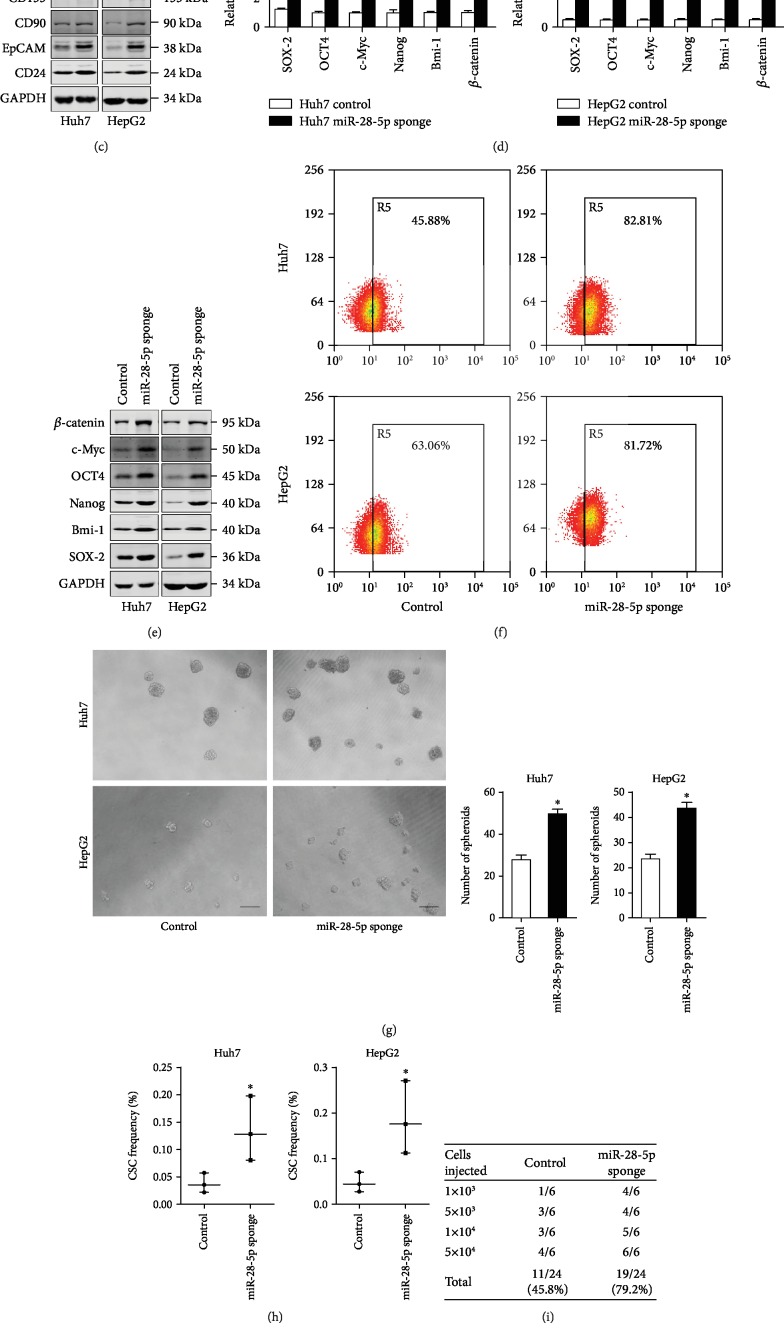
miR-28-5p interference derives liver CSC expansion. (a) Huh7 and HepG2 cells were infected with miR-28-5p sponge virus and control virus. The miR-28-5p interference effect was determined by RT-PCR assay (*n* = 3). (b) The mRNA expression of liver CSC markers in the miR-28-5p sponge and control HCC cells was checked by RT-PCR assay (*n* = 3). (c) The protein expression of liver CSC markers in the miR-28-5p sponge and control HCC cells was checked by western blot assay. GAPDH acted as a loading control. (d) The mRNA expression of stemness-associated genes in the miR-28-5p sponge and control HCC cells was checked by RT-PCR assay (*n* = 3). (e) The protein expression of stemness-associated genes in the miR-28-5p sponge and control HCC cells was checked by western blot assay. GAPDH acted as a loading control. (f) The EpCAM-positive cells in the miR-28-5p sponge and control HCC cells was determined by flow cytometry (*n* = 3). (g) The self-renewal ability of the miR-28-5p sponge and control HCC cells was compared by spheroid formation assay (*n* = 3). (h) *In vitro* limiting dilution assay of the miR-28-5p sponge and control HCC cells. The results are shown as a natural logarithm of the proportion of CSCs (*n* = 6). (i) The tumorigenicity of liver CSCs in the Huh7 miR-28-5p sponge and its control cells was compared by *in vivo* limiting dilution assay. Tumors were observed over 2 months; *n* = 6 for each group. (j) The protein expression of liver CSC markers and stemness-associated genes in above xenograft tumors was checked by western blot assay. GAPDH acted as a loading control. Data are represented as mean ± s.d.; ^∗^*P* < 0.05; two-tailed Student's *t*-test.

In order to explore the biological significance of miR-28-5p in liver CSCs, HCC cells were transfected with the miR-28-5p sponge virus. The miR-28-5p interference effect was confirmed by RT-PCR assay (Figure 2(a)). Downregulation of miR-28-5p in HCC cells notably increased the expression of CSC markers and stemness-related genes in hepatoma cells (Figures 2(b)–2(e)). Next, we found that the proportion of EpCAM in miR-28-5p knockdown hepatoma cells was upregulated (Figure 2(f)). Additionally, miR-28-5p interference hepatoma cells formed much more spheres compared with negative control cells (Figure 2(g)). *In vitro* limiting dilution assay found that miR-28-5p knockdown significantly increased the CSCs' frequency in hepatoma cells (Figure 2(h)). More importantly, *in vivo* limiting dilution assay indicted that miR-28-5p knockdown markedly upregulated the tumorigenesis capacity in hepatoma cells (Figure 2(i)). The protein of liver cancer stem markers and stemness-related genes in miR-28-5p knockdown xenograft tumors was also increased (Figure 2(j)).

### 4.3. miR-28-5p Inhibits Liver CSC Expansion

**Figure 3:**
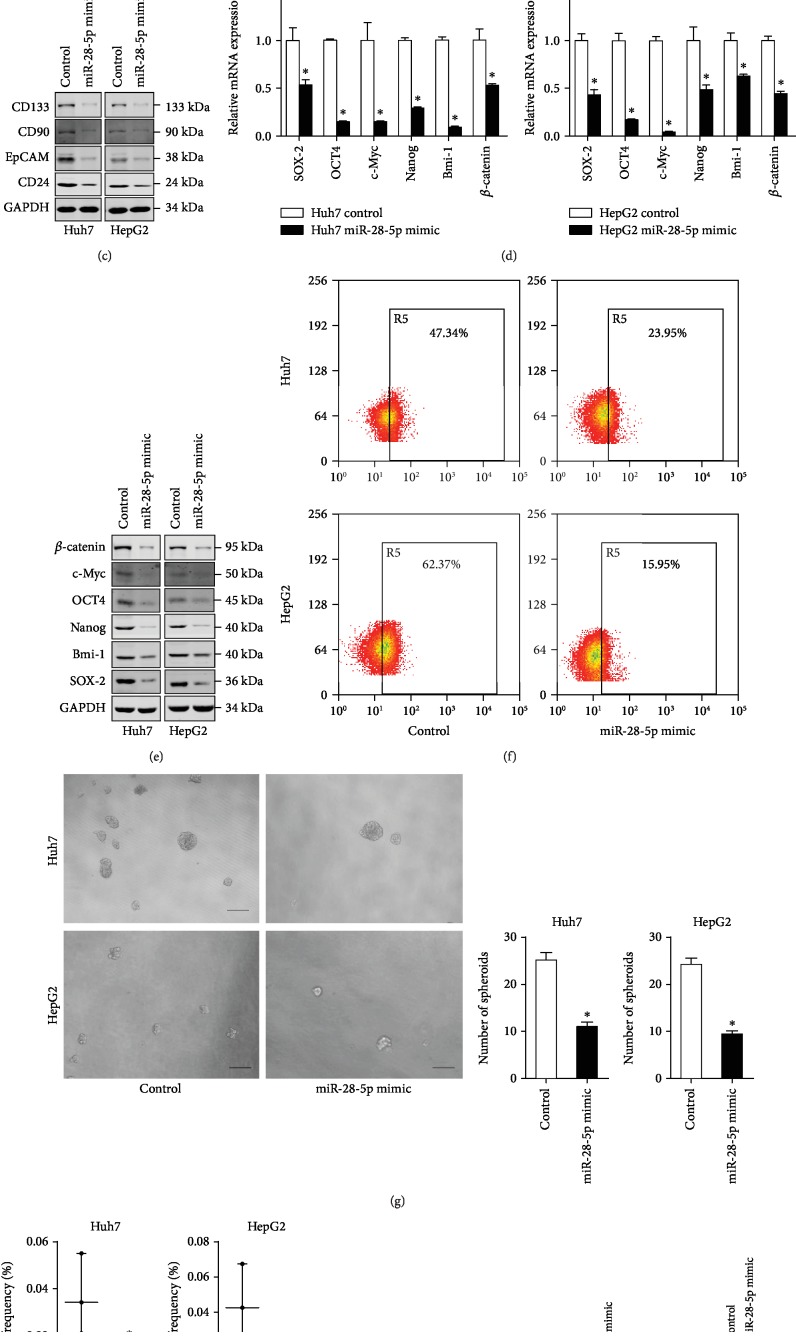
miR-28-5p overexpression suppresses liver CSC expansion. (a) Hepatoma cells were infected with the miR-28-5p mimic virus and control virus. The miR-28-5p overexpression effect was determined by RT-PCR assay (*n* = 3). (b) The mRNA expression of liver CSC markers in the miR-28-5p mimic and control HCC cells was checked by RT-PCR assay (*n* = 3). (c) The protein expression of liver CSC markers in the miR-28-5p mimic and control HCC cells was checked by western blot assay. GAPDH acted as a loading control. (d) The mRNA expression of stemness-associated genes in the miR-28-5p mimic and control HCC cells was checked by RT-PCR assay (*n* = 3). (e) The protein expression of stemness-associated genes in the miR-28-5p mimic and control HCC cells was checked by western blot assay. GAPDH acted as a loading control. (f) The EpCAM-positive cells in the miR-28-5p mimic and control HCC cells was determined by flow cytometry (*n* = 3). (g) The self-renewal ability of the miR-28-5p mimic and control HCC cells was compared by spheroid formation assay (*n* = 3). (h) *In vitro* limiting dilution assay of the miR-28-5p mimic and control HCC cells. The results are shown as a natural logarithm of the proportion of CSCs (*n* = 6). (i) The tumorigenicity of liver CSCs in the Huh7 miR-28-5p mimic and its control cells was compared by *in vivo* limiting dilution assay. Tumors were observed over 2 months; *n* = 6 for each group. (j) The protein expression of liver CSC markers and stemness-associated genes in the above xenograft tumors was checked by western blot assay. GAPDH acted as a loading control. Data are represented as mean ± s.d.; ^∗^*P* < 0.05; two-tailed Student's *t*-test.

To further explore the biological role of miR-28-5p in liver CSCs, HCC cells were transfected with miR-28-5p mimic virus. The miR-28-5p overexpression effect was confirmed by RT-PCR assay (Figure 3(a)). Upregulation of miR-28-5p in HCC cells dramatically decreased the expression of CSC markers and stemness-related genes in hepatoma cells (Figures 3(b)–3(e)). Next, we found that the proportion of EpCAM in miR-28-5p overexpression hepatoma cells was downregulated (Figure 3(f)). Additionally, miR-28-5p overexpression of hepatoma cells formed fewer spheres compared with negative control cells (Figure 3(g)). *In vitro* limiting dilution assay found that miR-28-5p overexpression significantly decreased the CSC frequency in hepatoma cells (Figure 3(h)). More importantly, *in vivo* limiting dilution assay indicated that miR-28-5p overexpression notably downregulated the tumorigenesis capacity in hepatoma cells (Figure 3(i)). The protein of liver cancer stem markers and stemness-related genes in miR-28-5p knockdown xenograft tumors was also reduced (Figure 3(j)). Collectively, the above results indicated that miR-28-5p inhibits liver CSC self-renewal and tumorigenesis.

### 4.4. IGF-1 Is a Direct Target of miR-28-5p in Liver CSCs

**Figure 4:**
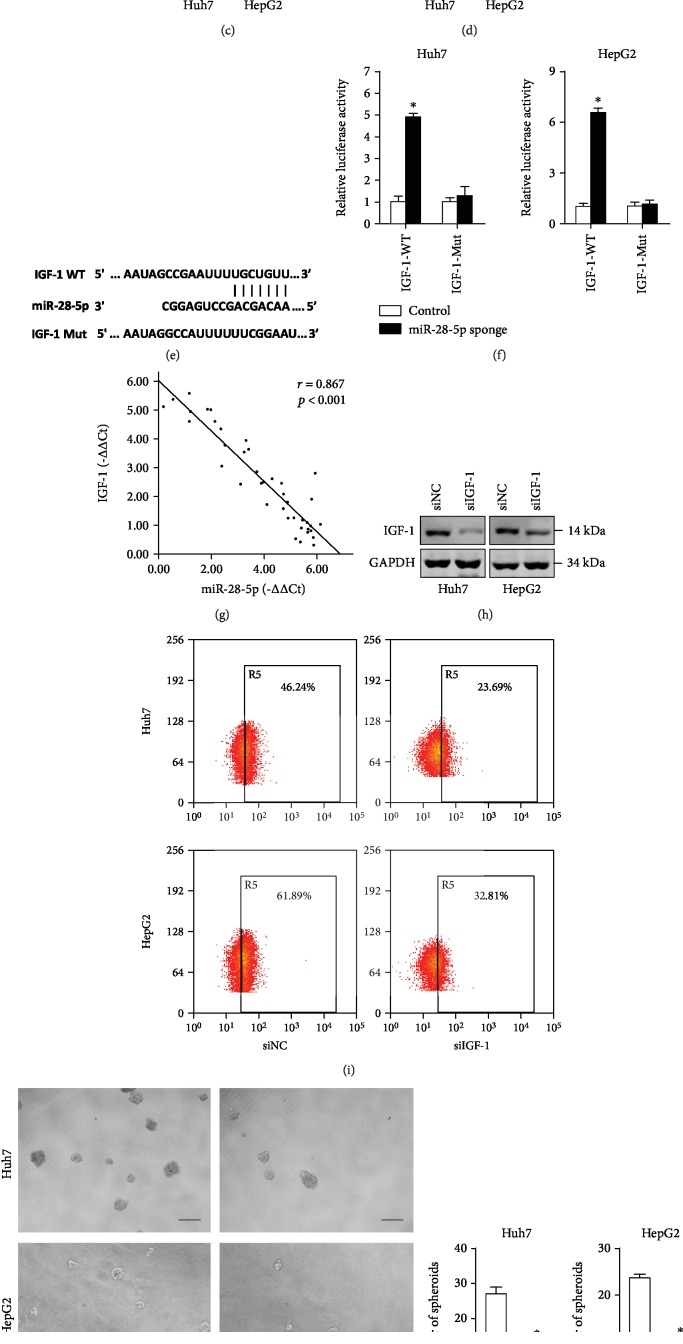
IGF-1 is required for miR-28-5p-mediated liver CSC expansion. (a) The mRNA expression of IGF-1 and IL-34 in the miR-28-5p sponge and control liver CSCs was determined by RT-PCR assay (*n* = 3). (b) The mRNA expression of IGF-1 and IL-34 in the miR-28-5p mimic and control liver CSCs was determined by RT-PCR assay (*n* = 3). (c) The protein expression of IGF-1 in the miR-28-5p sponge and control liver CSCs was checked by western blot assay. GAPDH acted as a loading control. (d) The protein expression of IGF-1 in the miR-28-5p mimic and control liver CSCs was checked by western blot assay. GAPDH acted as a loading control. (e) The miR-28-5p potential binding sites at the 3′-UTR of IGF-1 and the nucleotides mutated in the IGF-1-3′-UTR mutant. (f) Luciferase reporter assay was performed to detect the effect of the miR-28-5p sponge on the luciferase intensity controlled by 3′-UTR of IGF-1 (*n* = 3).(g) The correlation between the transcription level of miR-28-5p and IGF-1 in forty HCC tissues was determined by RT-PCR analysis. Data were normalized to U6 or *β*-actin as *Δ*Ct and analyzed by Spearman's correlation analysis. (h) Huh7 and HepG2 cells were transfected with IGF-1 siRNA or negative control and then subjected to western blot assay. GAPDH acted as a loading control. (i) The EpCAM-positive cells in siIGF-1 and control HCC cells were determined by flow cytometry (*n* = 3). (j) The self-renewal ability of siIGF-1 and control HCC cells was compared by spheroid formation assay (*n* = 3). (k) The miR-28-5p sponge and control HCC cells were transfected with siIGF-1 and siNC followed by flow-cytometric assay (*n* = 3). (l) The miR-28-5p sponge and control HCC cells were transfected with siIGF-1 and siNC followed by spheroid formation assay (*n* = 3). (m) The Huh7 miR-28-5p sponge and its control cells were transfected with siIGF-1 and siNC and then subjected to *in vivo* limiting dilution assay. Tumors were observed over 2 months; *n* = 6 for each group. Data are represented as mean ± s.d.; ^∗^*P* < 0.05; two-tailed Student's *t*-test.

It was reported that miR-28-5p targeted the 3′-UTRs of IL-34 and IGF-1 in HCC cells [21, 23]. So, we checked whether IL-34 and IGF-1 were also required for miR-28-5p-mediated liver CSC expansion. As shown in Figures 4(a) and 4(b), IGF-1 mRNA was upregulated in miR-28-5p overexpression liver CSCs and downregulated in miR-28-5p interference liver CSCs, while the IL-34 mRNA level was unchanged. Consistently, the IGF-1 protein level was also increased in miR-28-5p interference liver CSCs and decreased in miR-28-5p-overexpressing liver CSCs (Figures 4(c) and 4(d)). Bioinformatics analysis suggested that IGF-1 mRNA harbored a putative miR-28-5p binding site in its 3′-UTR (Figure 4(e)). To demonstrate the direct interaction between miR-28-5p and IGF1 mRNA, the luciferase reporter system containing the binding site (IGF1-3′-UTR-wt) or mutated site (IGF1-3′-UTR-mut) was transfected into miR-28-5p interference liver CSCs. The results showed that the luciferase activity in miR-28-5p knockdown liver CSCs was increased markedly compared with negative controls, while the miR-28-5p sponge did not affect the luciferase activity in the pGL3-IGF-1-mut vector (Figure 4(f)). Moreover, there was a significant negative correlation between miR-28-5p and IGF-1 mRNA expression in human HCC tissues (Figure 4(g)).

Next, we explore the biological function of IGF-1 in liver CSCs. HCC cells were infected with special IGF-1 siRNA, and the knockdown effect was determined by RT-PCR (Figure 4(h)). As expected, the proportion of EpCAM^+^ cells was downregulated in IGF-1 knockdown HCC cells (Figure 4(i)). Moreover, the self-renewal ability was also weakened in IGF-1 knockdown HCC cells (Figure 4(j)). These results showed that IGF-1 could promote liver CSC expansion. So, we treated miR-28-5p sponge HCC cells and its control cells with special IGF-1 siRNA and found that the difference in the proportion of liver CSCs, self-renewal ability, and tumorigenesis capacity between miR-28-5p knockdown and control hepatoma cells was diminished by special IGF-1 siRNA (Figures 4(k)–4(m)). Collectively, these results demonstrated that IGF-1 was required for miR-28-5p-mediated liver CSC expansion.

### 4.5. miR-28-5p Determines Sorafenib Response in HCC Cells

**Figure 5:**
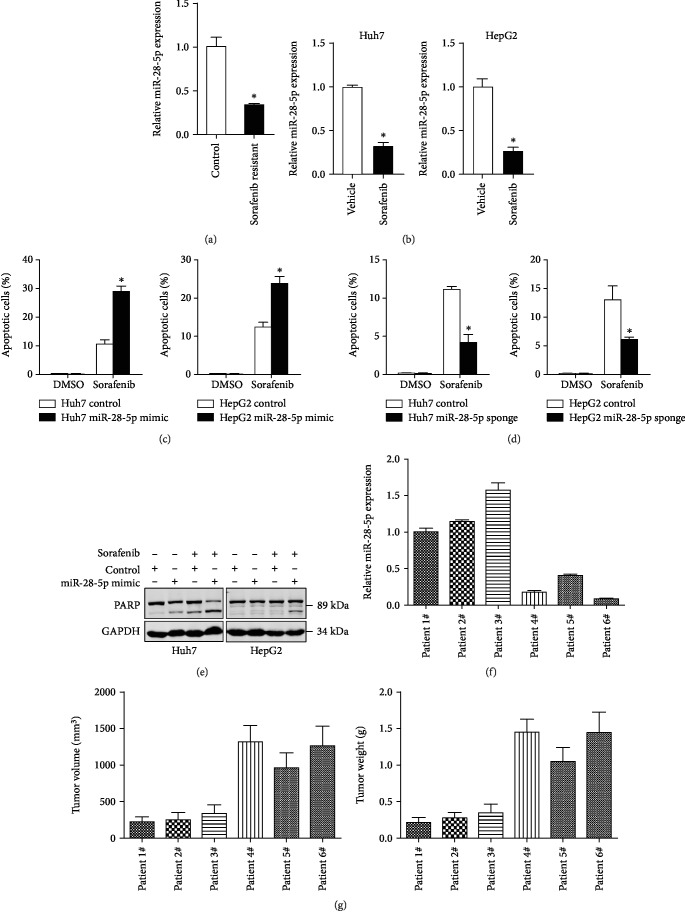
miR-28-5p knockdown HCC cells are more sensitive to sorafenib treatment. (a) The expression of miR-28-5p in sorafenib-resistant HCC xenografts was determined by RT-PCR assay (*n* = 3). (b) The expression of miR-28-5p in sorafenib-resistant HCC cells and control cells was determined by RT-PCR assay (*n* = 3). (c) The miR-29-5p mimic and control HCC cells were treated with sorafenib (10 *μ*M) for 48 hours and subjected to flow cytometry assay (*n* = 3). (d) The miR-29-5p sponge and control HCC cells were treated with sorafenib (10 *μ*M) for 48 hours and subjected to flow cytometry assay (*n* = 3). (e) The miR-29-5p mimic and control HCC cells were treated with sorafenib (10 *μ*M) for 48 hours and subjected to western blot assay. GAPDH acted as a loading control. (f) The expression of miR-28-5p in PDX primary tumors was determined by RT-PCR assay. (g) PDXs with low or high miR-28-5p levels in their primary tumors were treated with sorafenib (60 mg/kg body weight) or vehicle for 30 days (*n* = 5 for each group). The terminal tumor size and weight was showed. Data are represented as mean ± s.d.; ^∗^*P* < 0.05; two-tailed Student's *t*-test.

Increasing evidence shows that liver CSCs was involved in the resistance of cancers to targeted drugs and chemotherapeutic drugs [24]. We first checked the miR-28-5p expression in sorafenib-resistant HCC xenografts and cells. The results showed that miR-28-5p expression was dramatically downregulated in both sorafenib-resistant HCC xenografts and cells (Figures 5(a) and 5(b)). Next, we found that miR-28-5p overexpression led to the sensitivity of hepatoma cells to sorafenib-induced cell apoptosis (Figure 5(c)). Consistently, miR-28-5p knockdown led to the resistance of hepatoma cells to sorafenib-induced cell apoptosis (Figure 5(c)). Additionally, we also found that the protein level of PARP in miR-28-5p mimic hepatoma cells was significantly increased when they were exposed to the same doses of sorafenib when compared with control HCC cells (Figure 5(e)). Furthermore, we found that the PDXs derived from HCC tumors with low miR-28-5p levels were resistant to sorafenib treatment. In contrast, the PDXs derived from HCC tumors with high miR-28-5p levels were sensitive to sorafenib treatment (Figures 5(f) and 5(g)). Taken together, our results demonstrated that miR-28-5p might serve as a reliable predictor for sorafenib treatment.

## 5. Discussion

Hepatocellular carcinoma is the second leading cause of cancer mortality. Approximately 319,000 persons die from HCC every year in China, which accounts for 51% of the deaths from HCC worldwide [25]. Treatment for HCC includes resection, radiotherapy, chemotherapy, and biotherapy. Despite the recent progress in HCC prevention and intervention, the prognosis of HCC was also unsatisfactory due to the high rate of relapse and chemoresistance [26]. Numerous studies revealed that the poor prognosis of HCC was closely associated with the existence of liver CSCs [27]. So, it is urgent to find the molecular mechanism underlying liver CSC regulation for the sake of developing novel therapeutic strategies targeting CSCs. In the present study, for the first time, we clarify that miR-28-5p is reduced in liver CSCs and suppresses liver CSC self-renewal and tumorigenesis. We also demonstrated that the value of miR-28-5p plays an important role in the sensitivity of HCC cells to sorafenib.

It was reported that miR-28 participated in the regulation of several types of cancers, including gastric cancer, ovarian cancer, and prostate cancer [28–30]. In this study, miR-28-5p expression was found to be downregulated in sorted CD24- and EpCAM-positive primary HCC cells as well as primary HCC spheres. Furthermore, knockdown miR-28-5p in HCC cells upregulated liver CSC markers and promoted the self-renewal capacity and tumorigenicity of liver CSCs. On the contrary, overexpressed miR-28-5p in HCC cells downregulated liver CSC markers and inhibited the self-renewal capacity and tumorigenicity of liver CSCs. Previous studies found that miR-28-5p inhibited HCC cell metastasis via IL-34 and the insulin-like growth factor-1 (IGF-1) pathway [21, 23]. Thus, we sought to identify the downstream target genes of miR-28-5p and determine whether these genes accounted for miR-28-5p-mediated liver CSC expansion. We identified IGF-1 as a direct target for miR-28-5p in liver CSCs.

Insulin-like growth factor 1 (IGF-1) is a key regulator of programmed cell proliferation, differentiation, and apoptosis [31]. It was reported that abnormal activation of IGF-1 promoted tumor cell growth and metastasis [32], but the exact mechanism beneath IGF-1 activation in HCC remains vague. The PI3K/AKT and ERK/P38 signaling pathways were enhanced by IGF-1 signaling in a variety of tumorigenesis [33, 34]. In addition, IGF-1 was also reported to be involved in the modulation of CSCs [35]. In this study, we found that miR-28-5p downregulates IGF-1 expression through binding to its 3′-UTR in liver CSCs. Moreover, knockdown IGF-1 expression suppresses HCC cell self-renewal ability and downregulated liver CSC markers. Furthermore, special IGF-1 siRNA could abrogate the discrepancy of the self-renewal ability and tumorigenicity capacity between miR-28-5p knockdown liver CSCs and control cells. The correlation between miR-28-5p and IGF-1 is further validated in human HCC tissues.

Sorafenib is the first FDA-approved targeted drug for advanced HCC patients [36]. However, only a few HCC patients benefited from sorafenib treatment [37]. So, it is urgent to find a biomarker for sorafenib treatment in HCC. In this study, we find that miR-28-5p overexpression HCC cells are more sensitive to sorafenib-induced apoptosis and miR-28-5p interference HCC cells are more resistant to sorafenib-induced apoptosis. Furthermore, sorafenib PDX studies further demonstrate that a high miR-28-5p level in HCC patients can serve as a reliable predictor for sorafenib response.

Taken together, we demonstrate that miR-28-5p is reduced in liver CSCs, which in turn suppresses the self-renewal and tumorigenicity of liver CSCs. In addition, miR-28-5p inhibits liver CSC expansion through directly regulating IGF-1. In conclusion, our findings provide insight into the miR-28-5p/IGF-1 axis as a potential therapeutic target against liver CSCs and a potential predictor for sorafenib treatment of HCC patients.

## Data Availability

Data generated from the study are available from the corresponding authors on reasonable request.

## Supplementary Materials

Supplementary Materials Supplementary Table 1: clinicopathological features of 50 HCC patients. Supplementary Table 2: primer list. Supplementary Table 3: antibody list.
