# Supplementary material for: MicroRNA-28-5p Regulates Liver Cancer Stem Cell Expansion via IGF-1 Pathway
Source: Stem Cells Int. 2019 Dec 1;2019:8734362. doi: 10.1155/2019/8734362 (PMC6915025; doi:10.1155/2019/8734362)
Supplement: Supplementary Materials — Supplementary Table 1: clinicopathological features of 50 HCC patients. Supplementary Table 2: primer list. Supplementary Table 3: antibody list. [file 8734362.f1.docx]

**Supplementary Table**

**Supplementary Table 1. Clinicopathological features of 50 HCC patients**

| Characteristics |  |  | Total n=50 |
| --- | --- | --- | --- |
| Age(year) | ≤50 |  | 36 |
|  | >50 | | 14 |
| Gender | Male | | 46 |
|  | Female | | 4 |
| HBsAg | Positive | | 45 |
|  | Negative | | 5 |
| AFP(μg/L) | ≤400 | | 38 |
|  | >400 | | 12 |
| Tumor size(cm) | ≤5 | | 9 |
|  | >5 | | 41 |
| Tumor number | Single | | 28 |
|  | Multiple | | 22 |
| Portal vein tumor thrombus | Yes | | 6 |
|  | No | | 44 |
| Pathological satellite | Yes |  | 16 |
|  | No |  | 34 |
| BCLC stage | A | | 20 |
|  | B or C | | 30 |
| TNM | I-II |  | 19 |
|  | I-II |  | 31 |

**Supplementary Table 2. Primer List.**

| **Gene** | **Forward primer (5’-3’)** | **Reverse primer (5’-3’)** | |
| --- | --- | --- | --- |
| β-actin(Human) | Forward (5*′*- 3*′*) | GGCCCAGAATGCAGTTCGCCTT | |
|  | Reverse (5*′*- 3*′*) | AATGGCACCCTGCTCACGCA | |
| CD133(Human) | Forward (5*′*- 3*′*) | AGAGGAAGCCGCAAC | |
|  | Reverse (5*′*- 3*′*) | CTGGCTCGTGAATTATTTAT | |
| CD24(Human) | Forward (5*′*- 3*′*) | GCAAACAGATGTGTTCTTAAT | |
|  | Reverse (5*′*- 3*′*) | TCATCCCTAAGATCAAGTTT | |
| CD90(Human) | Forward (5*′*- 3*′*) | GAATACGGAAATGGATTAAG | |
|  | Reverse (5*′*- 3*′*) | GTATTCATTTCCTCTGGTCT | |
| EpCAM(Human) | Forward (5*′*- 3*′*) | | CGCAGCTCAGGAAGAATGTG |
|  | Reverse (5*′*- 3*′*) | | TGAAGTACACTGGCATTGACGA |
| SOX2(Human) | Forward (5*′*- 3*′*) | | TGGAGAAGGAATGGTCCACTTC |
|  | Reverse (5*′*- 3*′*) | | GGATAAGTACACGCTGCCCG |
| OCT4(Human) | Forward (5*′*- 3*′*) | | ATGTGCGCGTAACTGTCCAT |
|  | Reverse (5*′*- 3*′*) | | CTGCAGTGTGGGTTTCGGGCA |
| Nanog(Human) | Forward (5*′*- 3*′*) | | AATACCTCAGCCTCCAGCAGATG |
|  | Reverse (5*′*- 3*′*) | | TGCGTCACACCATTGCTATTCTTC |
| c-Myc(Human) | Forward (5*′*- 3*′*) | | CCCTCCACTCGGAAGGACTA |
|  | Reverse (5*′*- 3*′*) | | GCTGGTGCATTTTCGGTTGT |
| Bmil-1(Human) | Forward (5*′*- 3*′*) | | TGGAGAAGGAATGGTCCACTTC |
|  | Reverse (5*′*- 3*′*) | | GTGAGGAAACTGTGGATGAGGA |
| β-catenin(Human) | Forward (5*′*- 3*′*) | | CGCTGGATTTTCAAAACAGT |
|  | Reverse (5*′*- 3*′*) | | CTGAGGAGCAGCTTCAGTCC |
| IGF-1(Human) | Forward (5*′*- 3*′*) | | ATGTTCCCCCAGCTGTTTCC |
|  | Reverse (5*′*- 3*′*) | | ATTCCATTGCGCAGGCTCTA |
| IL-34(Human) | Forward (5*′*- 3*′*) | | GAGACCGAGTCTTGGCACG |
|  | Reverse (5*′*- 3*′*) | | TGGGTGACGCTTTCTCTGAT |

**Supplementary Table 3. Antibody List.**

| **Antigens** | **Manufacturer** | **Application** |
| --- | --- | --- |
| IGF-1 | Abcam, USA | 1:500 for WB |
| CD133 | Abcam, USA | 1:500 for WB |
| EpCAM | Proteintech, China | 1:500 for WB |
| CD90 | Abcam, USA | 1:500 for WB |
| CD24 | Abcam, USA | 1:500 for WB |
| SOX-2 | Cell Signaling Technology, Beverly, MA | 1:500 for WB |
| OCT4 | Cell Signaling Technology, Beverly, MA | 1:500 for WB |
| c-Myc | Cell Signaling Technology, Beverly, MA | 1:500 for WB |
| Nanog | Cell Signaling Technology, Beverly, MA | 1:500 for WB |
| Bmi-1 | Cell Signaling Technology, Beverly, MA | 1:500 for WB |
| β-catenin | Cell Signaling Technology, Beverly, MA | 1:500 for WB |
| PARP | Cell Signaling Technology, Beverly, MA | 1:500 for WB |
| GAPDH | Santa Cruz Biotechnology, CA | 1:5000 for WB |
